# Retinol and Retinyl Palmitate in Foetal Lung Mice: Sexual Dimorphism

**DOI:** 10.1155/2013/760305

**Published:** 2013-01-13

**Authors:** Olga Carvalho, Carlos Gonçalves

**Affiliations:** Histology and Embryology Institute, Faculty of Medicine, University of Coimbra, Rua Larga, 3004-504 Coimbra, Portugal

## Abstract

In this work, we evaluate the lung retinoids content to study the possible difference between male and female mice during prenatal development and to comprehend if the vitamin A metabolism is similar in both genders. The study occurred between developmental days E15 and E19, and the retinol and retinyl palmitate lung contents were determined by HPLC analysis. We established two main groups: the control, consisting of foetuses obtained from pregnant females without any manipulation, and vitamin A, composed of foetuses from pregnant females submitted to vitamin A administration on developmental day E14. Each of these groups was subdivided by gender, establishing the four final groups. In the lung of control group, retinol was undetected in both genders and retinyl palmitate levels exhibited a sexual dimorphism. In the vitamin A group, we detected retinol and retinyl palmitate in both genders, and we observed a more evident sexual dimorphism for both retinoids. Our study also indicates that, from developmental day E15 to E19, there is an increase in the retinoids content in foetal lung and a gender difference in the retinoids metabolism. In conclusion, there is a sexual dimorphism in the lung retinoids content and in its metabolism during mice development.

## 1. Introduction

Vitamin A has been regarded as a major contributor in the differentiation and maturation of the lung [1–3], and there is no doubt that retinoids, especially retinoic acid, are essential for the lung development [4]. 

Until now, little is known about the acquisition and use of stored retinoids, but functionally they are involved in lung differentiation and maturation [2, 3], surfactant production [5, 6], inducing the formation of alveolar septa [7–9], cell differentiation [1, 2, 10, 11], elastin synthesis and deposition [12–17], homeostasis and lung repair [18, 19], and alveolar regeneration capacity [20, 21]. 

Some studies showed the potential usefulness of retinoids in reducing the incidence of bronchopulmonary dysplasia (BPD) in newborns, because indirectly retinoic acid is able to inhibit the effect of glucocorticoids that are often used to treat this pathology [22, 23]. The administration of vitamin A to premature newborns with low birth weight subjected to mechanical ventilation promotes the regeneration of lung injury by reducing the morbidity associated with BPD [22, 23]. Administration of a vitamin A supplement 48 hours after birth significantly reduced the mortality of newborns during the first 3-4 months of life, and the greatest benefit occurs in children with low birth weight birth [24]. Kennedy and collaborators treated premature infants with vitamin A and observed a reduction in the incidence of bronchopulmonary diseases and a reduction in the mortality [25]. Retinoids also restricted inflammation by reducing the cell death and extracellular matrix degradation [26, 27]. 

During foetal development, lung accumulates retinoids, in particular retinol and retinyl esters [16, 28], but the biologically active molecule is retinoic acid (RA) [2, 3]. In fact, the retinoic acid deficiency during pregnancy causes severe defects in lung development, including lung hypoplasia and agenesis [29–31]. 

RA is generated by a series of oxidative reactions that convert retinol to retinaldehyde and ultimately to the active form retinoid acid [32]. The pleiotropic effects of retinoic acid are due to the variety of RA isoforms, polymorphism of the receptors RAR and RXR, and to the possibility that RXR have to form heterodimers with other receptors [30, 32].

The retinoids metabolism and homeostasis is controlled by dietetic availability, but also by an accurate mechanism of absorption, transportation, and reserve mobilization [32–34]. About 75% to 95% of retinoids are in the liver stellate or Ito cells, but it can also be deposited in other organs such as kidney, intestine, lung, and eye, although in the adult this storage is minimal when compared to the total quantity in the liver [33]. 

In the plasma, retinol circulates bounded to a complex formed by two proteins, retinol binding protein (RBP) and transthyretin (TTR) [35, 36]. Inside the cell retinol is complexed with the cellular retinol binding protein—CRBP—type I or type II, and free retinol is almost undetectable [37]. The complexed retinol can have different targets, that is, transformed into AR; excreted to the extracellular medium, if there is no immediate need for retinoids; or accumulated in the form of retinyl esters [33, 34]. 

The lung cells that store retinol are similar to the liver Ito cells [38], but in the developing lung, these cells are the lipid-containing interstitial cells (LICs) [16, 39]. The LICs are present during alveogenesis [15, 40], period in which the number of this cells increased [26], and are the main producers of tropoelastin [15], which under the action of RA increase the synthesis and deposition of this protein [16]. 

In the embryonic stage of lung development, there is abundant synthesis and use of the RA by the primitive foregut, which demonstrates their direct involvement in the lung primordial bud formation [29, 41–43]. 

During the branching morphogenesis, retinoic acid signal remains low and the levels of enzyme RALDH-2 are locally controlled, being concentrated in the regions with less branching activity [43]. With the beginning of lateral buds, a RA proximal-distal gradient is established, with lower concentration in the distal mesenchyme near the sites of prospective budding [43]. The mesenchyme inactivation of retinoic acid signal allows the expression of FGF-10, which is the major responsible in the branching process [44, 45]. The inhibitory effect of RA on FGF-10 involves other molecules present in the epithelium and mesenchyme. RA activates the SHH protein, which inhibits the expression of Fgf-10 via PTCH pathway [44] or control the TGF-*β* activity, which acts in the local expression of FGF-10 [45]. 

At the alveolar stage, retinoic acid is functionally involved in several processes, already mentioned above, and during this stage we can observe an increase in expression of retinoic acid m-RNA, RARs receptors, and CBRP protein, which is consistent with the hypothesis that the endogenous RA contributes to the pulmonary development [2, 14, 15, 19]. 

Considering that retinoids are important in the lung morphogenesis mechanism and pulmonary function, that male children have a higher risk of neonatal death when compared to female children, and that the majority of diseases in neonatal life occur in the respiratory system, we thought it is pertinent and important to study possible existence of a sexual dimorphism in the lung retinoids content during prenatal life.

## 2. Material and Methods 

### 2.1. Experimental Model

In our experimental model, adult (60–70 days) male and female CD1 mice from “Charles River Laboratories-Research Models and Services,” were housed in the usual conditions, that is, 21°C temperature, 8/12 hours light/dark cycle, standard pellets, and water *ad libitum*. Mating was carried out under polygamous conditions, and in each male compartment we placed 3 females for a period of 16 hours. Developmental day 1 was determined based on the presence of a sperm plug and pregnancy was monitored (birth occurred between developmental days 19 and 20). 

All procedures involving animals were approved by the scientific committee, supervised by a Federation of European Laboratory Animal Science Association- (FELASA-) trained scientist, and conformed to regulation of Portuguese law (Portaria 1005/92) based on European Union Laboratory Animal Experimentation Regulations.

We establish two main groups, control and the vitamin A foetus that were subdivided according to the gender, making a total of four experimental groups and a total of 600 foetus. The pregnant mice from group vitamin A were submitted to an injection of 150 *μ*L of Aerovit (45000 UI) on the day E14 and no manipulation was made in the control pregnant mice. 

The euthanasia of pregnant mice was performed with an intramuscular injection ketamine/xylazine solution, at a dose of 0.05 mg/g body weight. After sternotomy, 0.3 mL of a sodium heparin solution at a concentration of 5000 UI/mL was injected by an intracardiac catheter. After the spread of anticoagulant into the general circulation of the mother, we collected the foetuses by Caesarean section and immediately placed in a saline solution. 

The lung samples were collected from developmental day E15 to day E19, and frozen at −80°C, until the high liquid pressure chromatography (HPLC) analysis was made, to quantify the retinol and retinyl palmitate levels.

The foetal sex was determined by light microscopy observation of the developmental gonads. 

According to the developmental day, the number of lung samples was different due to the lung size difference, but the total mass of lung was similar in all developmental days and always obtained from at least four different litters. 

All results were analysed with the program Statview 5.0, using the student *t*-test (paired) to compared groups, with a statistically significant value of *P* < 0.0001.

### 2.2. High-Performance Liquid Chromatography (HPLC)

#### 2.2.1. Equipment

Lung retinol and retinyl palmitate were determined by HPLC, using a programmable liquid chromatographic system “Gilson, Unipoint, V1.9 system software.” The UV/Vis detector was a Gilson, 151 equipment and the readings were done with a wavelength of 325 nm and with sensibility adjusted for 0.002 aufs. The HPLC column was a reversed-phase “Waters-Spherisorb, ODS2” stainless steel column (25 cm × 4.6 mm I.D.) from Waters associated, Inc., Milford, MA, USA. 

In our system, 100% methanol was used as the mobile phase to separate retinol, retinyl palmitate, and retinyl acetate (added to the sample as an internal standard). The flow rate was always 2.5 mL/min and the system was adjusted to elute retinol at 1.8 min and retinyl palmitate at 11 min. 

#### 2.2.2. Sample Extraction Method

750 *μ*L of the internal standard solution (2.5 *μ*g/mL retinyl acetate, spiked with 1 *μ*g/mL retinol and 4.5 *μ*g/mL retinyl palmitate) and 3 mL of chloroform/methanol solution (2 : 1), containing 50 *μ*g/mL of butylated hydroxy-toluene were added to the initial lung samples. After complete homogenization, the samples were centrifuged for 10 min (2000 g) to separate the layers. The top layer was removed and the residue was preserve at −20°C for total protein quantification. To 2 mL of the top layer we added 400 *μ*L of potassium chloride (0.37% p/v) and centrifuged for 5 min (2000 g). The upper part was neglected and the lower layer was totally evaporated under an argon flow. The residue was dissolved in 1 mL chloroform/methanol solution (1 : 1), and after filtered (0.2 *μ*m) was prepared for chromatographic analysis. 

#### 2.2.3. Total Protein Quantification

All these procedures were made using the protein kit, based on the Lowry's reaction, and also used to establish the calibration curve, with the final protein concentrations of 50, 100, 200, 300, and 400 *μ*g/mL. 

To begin, we diluted our sample in 1 mL distilled water, homogenized, and added 0.1 mL of sodium deoxycholate (0.15%) for 10 min. After that, 0.1 mL of trichloroacetic acid (70% p/v) were added and centrifuged for 8 min (10000 rpm). The supernatant was neglected and the “pellets” were dissolved in 0.1 mL of Lowry reagent for 20 min and then added Folin & Fenol Ciocalteu to give colour to the samples (30 min). All these procedures were done at room temperature.

The absorbance was determined using the spectrophotometer, in a wavelength of 750 nm, and using the calibration curve established in the beginning of this methodology. 

### 2.3. Chemicals

All solvents used have high purity and were acquired from Sigma, Analar and Panreac. The retinol, retinyl palmitate, and retinyl acetate were acquired from Sigma and their reference were, respectively, R7632, R3375, and R4632. 

The Micro-Lowry Peterson's kit used was acquired from Sigma P5656.

## 3. Results

During the five developmental days studied (E15 to E19), retinol was not detected in the lungs of males and females of the control group.

**Figure 1:**
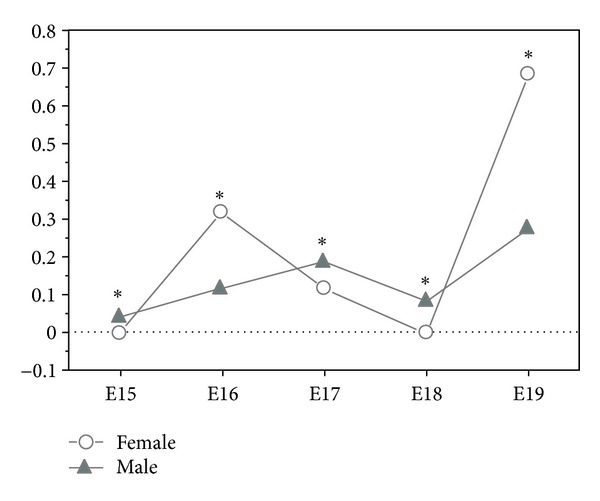
Lung retinol in the male and female foetuses of vitamin A group (ng/*μ*g protein), from developmental days E15 to E19 (all SD values were ≤0.007 and SE ≤ 0.002; *P < 0.0001 statistically significant).

On the contrary, in female and male foetuses of the vitamin A group, retinol was quantified, and variations were observed throughout the developmental (Figure 1). We only detected lung retinol in females on developmental days E16, E17, and E19, whose values were 0.315 ng, 0.117 ng, and 0.684 ng, respectively. In the males, retinol was detected in all developmental days, observing that in the three initial days the values gradually increase, that is, 0.042 ng on day E15, 0.115 ng on day E16, and 0.188 ng on day E17. On day E18 values dropped to 0.082 ng, and increased on day E19 to 0.273 ng (Figure 1). Both genders had the highest retinol value on developmental day E19 (Figure 1).

The comparative study between males and females of vitamin A group showed statistically significant (*P* < 0.0001) differences in all developmental days (Figure 1). On developmental days E16 and E19, females had more retinol than males, but on the remaining three days, males showed a higher value than females (Figure 1). 

**Figure 2:**
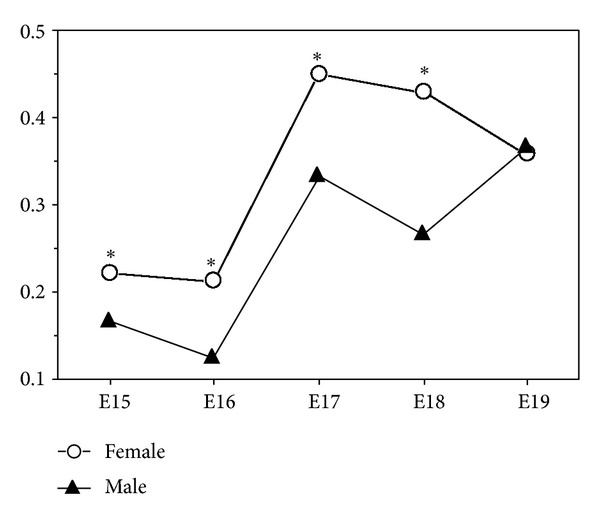
Lung retinyl palmitate in the male and female foetuses of control group (ng/*μ*g protein), from developmental days E15 to E19 (all SD values were  ≤0.003 and SE = 0.001; *P < 0.0001 statistically significant).

From developmental days E15 to E19, retinyl palmitate was detected in the lungs of males and females of control group (Figure 2). Females retinyl palmitate values were 0.220 ng on day E15, 0.212 ng on day E16, increasing to 0.449 ng on E17. In the following days, retinyl palmitate value decreased to 0.430 ng on day E18 and to 0.359 ng on day E19. In the first three developmental days the male's retinyl palmitate values were always lower than females, with 0.167 ng on day E15, 0.126 ng on day E16, and 0.334 ng on day E17. On day E18 retinyl palmitate decreased to 0.266 ng and increased to 0.366 ng on day E19 (Figure 2). The highest retinyl palmitate value in the male foetuses was observed on developmental day E19 and in the female on day E17 (Figure 2). 

The comparative study between male and female foetuses of control group evidence that from day E15 to E18, males had lower levels of retinyl palmitate than females, and that these differences were statistically significant (*P* < 0.0001) (Figure 2). On day E19 both genders had very similar retinyl palmitate content (Figure 2).

**Figure 3:**
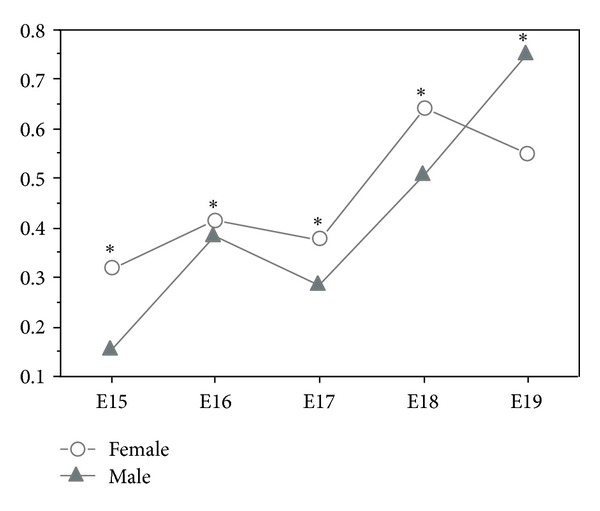
Lung retinyl palmitate in the male and female foetuses of vitamin A group (ng/*μ*g protein), from developmental days E15 to E19 (all SD values were ≤0.003 and SE = 0.001; *P < 0.0001 statistically significant).

In the vitamin A group, we quantify retinyl palmitate between the developmental days E15 to E19 in both genders (Figure 3). In the females, values were 0.320 ng retinyl palmitate on day E15, 0.414 ng on day E16, decreasing to 0.377 ng on day E17. In the next day (E18) the value increased to 0.639 ng and decreased to 0.549 ng on day E19. For the male's foetuses, retinyl palmitate values were 0.154 ng on day E15, 0.386 ng on day E16 and 0.287 ng on day E17. On the following days, E18 and E19, the values increased to 0.507 ng and 0.753 ng, respectively (Figure 3). The highest retinyl palmitate value in the male foetuses was observed on developmental day E19 and in the females on day E18 (Figure 3). 

In the comparative analysis between male and female foetuses of vitamin A group we observed that from developmental day E15 to E18, males had less retinyl palmitate than females (Figure 3). On day E19, male foetuses presented more retinyl palmitate than females. All these differences were statistically significant (*P* < 0.0001) (Figure 3).

**Figure 4:**
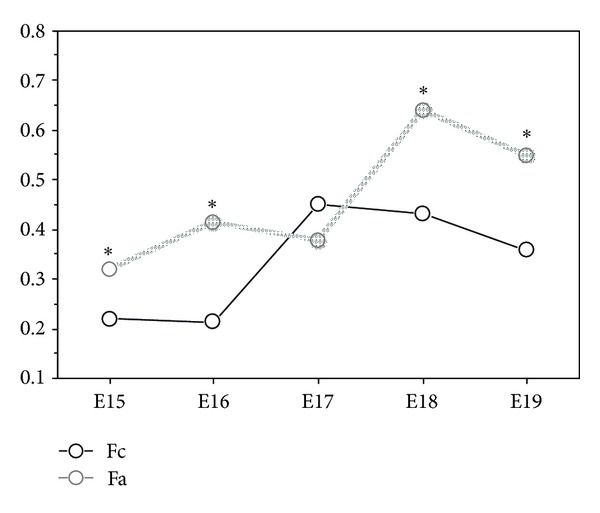
Lung retinyl palmitate in the female of control (Fc) and vitamin A (Fa) groups (ng/*μ*g protein), from developmental days E15 to E19 (*P < 0.0001 statistically significant).

When we compare the lung retinyl palmitate content between the control females and vitamin A females, we observed that on developmental days E15, E16, E18, and E19, control females have lower levels of retinyl palmitate, and that all these differences were statistically significant (*P* < 0.0001) (Figure 4).

On day E17, control females foetuses showed higher levels of retinyl palmitate, but this difference was not statistically significant (Figure 4). 

**Figure 5:**
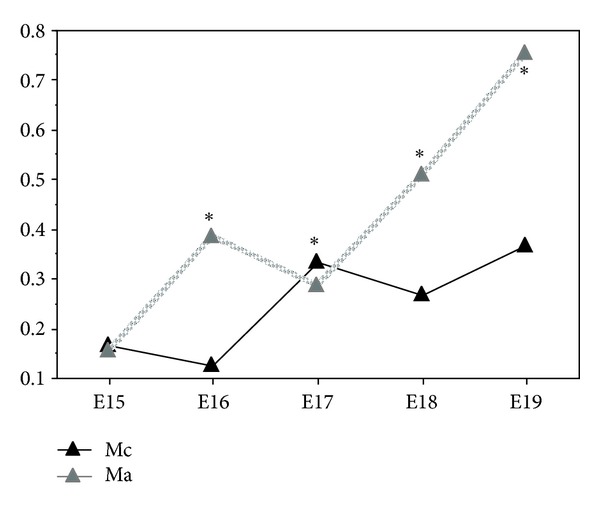
Lung retinyl palmitate in the male of control (Mc) and vitamin A (Ma) groups (ng/*μ*g protein), from developmental days E15 to E19 (*P < 0.0001 statistically significant).

The comparative analysis between males of control and vitamin A groups, evidence that on days E16, E18 and E19 the retinyl palmitate lung content was lower in the control group. On day E15 the retinyl palmitate value was very similar for both groups, but on day E17, the male foetuses of the control group had more retinyl palmitate (Figure 5). 

The differences observed between groups were statistically significant on developmental days E16, E17, E18, and E19 (*P* < 0.0001) (Figure 5).

## 4. Discussion 

In this work, retinol was not detected in the lung of male and female foetuses of control group, between developmental days E15 to E19. The results do not allow us to consider the complete absence of lung retinol, because it could be present in such a small amount that our methodology could not quantify. 

Vitamin A group had lung retinol during the developmental days studied, but some content variations were observed according to foetal gender. Male foetuses accumulated retinol during all developmental days, while females only stored on developmental days E16, E17, and E19. The lung retinol amount in the male suffered fewer oscillations than female's, and with the exception of day E17, male accumulate retinol throughout developmental. The higher retinol value was observed on day E19 for both genders. These results evidence a gender difference throughout the studied days, which can be understood as a sexual dimorphism, more evident on developmental day E19, where females showed more than twice the value of males.

We also observed that during the developmental days E15 to E19, the retinol metabolism was different for each gender. The ability to metabolize and store retinol was not equal for both sexes, as well as the retinol quantity stored.

In the lungs of the control group, retinyl palmitate was quantified in both genders, with variations throughout developmental according with the foetal gender. Results showed that between developmental days E15 to E18, the variations in the retinyl palmitate content were similar in both gender, however, retinyl palmitate levels were very different between the two genders. From developmental days E15 to E18, females had more retinyl palmitate than males, but on day E19, this values became equivalent. The retinyl palmitate peak was on day E19 for males and on day E17 for females. We can conclude that during days E15 to E18, control group exhibit a sexual dimorphism, and that females have greater capacity to accumulate retinyl palmitate in the lung. 

In the vitamin A group, retinyl palmitate was quantified, and variations throughout developmental were observed in accordance with the foetal gender. Although both genders have different retinyl palmitate lung content, we observed similar variations from developmental days E15 to E18. Females have higher retinyl palmitate levels than males, from days E15 to E18, and on day E19, the gender differences remained, but with male's displaying higher values when compared with females. 

With these results we can conclude that the vitamin A group also exhibits a sexual dimorphism, that is more evident and occurs during all developmental days studied. This accentuated dimorphism probably results from the gender different capacity to metabolize lung retinoids. 

Our results also showed that between developmental days E15 to E18, females of control and vitamin A groups, accumulate more retinyl palmitate than males from their respective groups. On day E19, control and vitamin A females decreased the level of lung retinyl palmitate, whereas the males from control and vitamin A groups increased this value. These data clearly demonstrate that female and male have different retinoids metabolism and that female are capable of accumulating more retinoids in the lung when compared with male. 

Finally, our study also showed that from developmental days E15 to E19, there is a trend to increase the retinoids concentration in the foetal lung mice. Other studies with foetal lung rat, detected retinyl esters on the developmental day E14, and after day E15, their concentration increased rapidly, reaching a peak value around day E18, followed by a decline to reach the lowest value on the first days of postnatal life [14, 15, 39]. 

## 5. Conclusions

Understanding the lung morphogenesis and knowledge of lung structural differentiation process and action of certain factors during prenatal life, are of extreme importance to the lung, an organ that completes its structural and functional maturation in the postnatal life. 

Several studies have shown that retinoids are important in the lung morphogenesis mechanism and in the expression of a number of components that are essential to the structure and pulmonary function. 

Our study showed the existence of a sexual dimorphism in the lung retinoids contents during prenatal life in the mice. We observed that the administration of vitamin A during the developmental development, emphasize the differences between genders, fact that could be explained by the different ability to metabolize retinoids presented by the male and female foetuses.
